# HDL Cholesterol Level Is Associated with Contrast Induced Acute Kidney Injury in Chronic Kidney Disease Patients Undergoing PCI

**DOI:** 10.1038/srep35774

**Published:** 2016-10-24

**Authors:** Hoon Suk Park, Chan Joon Kim, Byung-Hee Hwang, Tae-Hoon Kim, Yoon Seok Koh, Hun-Jun Park, Sung-Ho Her, Sung Won Jang, Chul-Soo Park, Jong Min Lee, Hee-Yeol Kim, Doo Soo Jeon, Pum-Joon Kim, Ki-Dong Yoo, Kiyuk Chang, Dong Chan Jin, Ki-Bae Seung

**Affiliations:** 1 Nephrology Division, Department of Internal Medicine, College of Medicine, The Catholic University of Korea, Republic of Korea; 2 Cardiology Division, Department of Internal Medicine, College of Medicine, The Catholic University of Korea, Republic of Korea

## Abstract

Chronic kidney disease (CKD) is a significant risk factor for contrast induced acute kidney injury (CI-AKI) after percutaneous coronary intervention (PCI). This study included 1592 CKD patients extracted from a prospective multicenter, all comer-based registry of patients undergoing PCI. In multivariate logistic analysis for CI-AKI development, a significant linear trend was observed between the quartiles of HDL-C (quartile 1 vs. 2: odds ratio [OR], 0.716; 95% confidence interval [CI], 0.421–1.219; quartile 1 vs. 3: OR, 0.534; 95% CI, 0.301–0.947; quartile 1 vs. 4: OR, 0.173; 95% CI, 0.079–0.377; *P* for trend < 0.001). HDL-C quartiles were also negatively correlated with the incidence of CI-AKI; 19.0%, 12.1%, 8.7%, and 3.7% for quartile 1(Q1) (<34 mg/dL), Q2 (34–40 mg/dL), Q3 (40–48 mg/dL), and Q4 (>48 mg/dL) respectively (*P* < 0.001 overall and for the trend). Multivariate Cox regression analysis for the long term mortality, the highest HDL-C quartile was associated with decreased mortality compared with the lowest HDL-C quartile (hazard ratio [HR] 0.516, 95% CI, 0.320–0.832, *P* = 0.007). Our study suggests more intensive strategies should be considered for preventing CI-AKI in CKD patients with low serum HDL-C level who is planned for PCI.

As both the number of elderly people and the prevalence of diabetes mellitus (DM) continue to rise, the incidence and prevalence of chronic kidney disease (CKD) increase rapidly^1^,^2^. These CKD patients are the population at risk of cardiovascular disease such as ischemic heart disease (IHD) and cerebrovascular accident (CVA)^3^. Endovascular procedure based on angiography using contrast media has been increasingly employed in cardiovascular patients because it is less invasive than open surgery and minimizes the procedure-related risk that may be related to procedure itself^4^,^5^. However, contrast media are nephrotoxic and sometimes lead to irreversible renal damage.

Contrast-induced acute kidney injury (CI-AKI) after primary percutaneous coronary intervention (PCI) is known to be associated with the increase in mortality during admission and the development of major cardiac adverse events even after discharge^6^,^7^. Older age, anemia, contrast volume, peri-procedural hemodynamic instabilities such as the use of intra-aortic balloon pump, initial lower left ventricular ejection fraction (LVEF), DM and acute hyperglycemia have been also reported as the risk factors for CI-AKI but the degree of the underlying CKD is the most important determinant for CI-AKI development^8^,^9^,^10^,^11^. Only limited data are available in terms of risk factors of CI-AKI in CKD patients. We conducted this observational cohort study to find the risk factors for CI-AKI in the CKD patients undergoing PCI.

## Results

### Baseline demographic, clinical and laboratory profiles

Among the 1592 patients in this study, 193 (12.1%) patients developed CI-AKI. As shown in Table 1, The CI-AKI group was older and had a higher prevalence of DM and CVA. BMI, LVEF, hemoglobin, albumin and pre-procedural Modification of Diet in Renal Disease estimated glomerular filtration rate (MDRD eGFR) levels were lower, while the level of pre-procedural plasma glucose, uric acid and phosphorus were higher in the CI-AKI group. In the lipid profile, only high density lipoprotein cholesterol (HDL-C, median: 35.0 mg/dL, interquartile range [IQR]: 29.0 to 41.0 mg/dL vs. median: 41.0 mg/dL, IQR: 34.0 to 49.0 mg/dL, *P* < 0.001) level were lower in the CI-AKI group.

### Predictors of the development of CI-AKI and long term mortality

**Figure 1:**
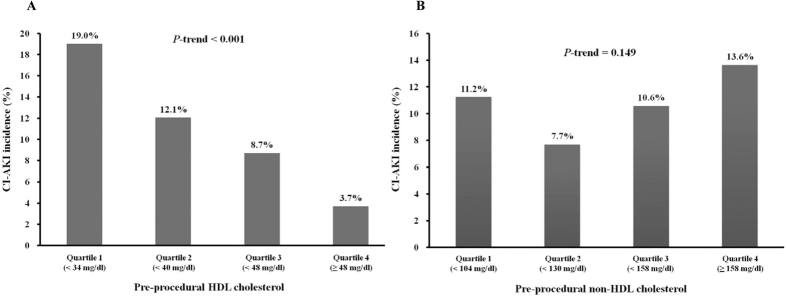
(**A**) Relationship between the pre-procedural HDL cholesterol level and CI-AKI. The association between HDL cholesterol level and the percentage of patients with CI-AKI was significant (*P* < 0.001 overall and for the trend). (**B**) Relationship between the pre-procedural non-HDL cholesterol level and CI-AKI. The association between pre-procedural non-HDL cholesterol level and the percentage of patients with CI-AKI was not significant (*P* = 0.149 overall and for the trend).

Univariate and multivariate logistic regression analyses were performed to assess the effects of variables on CI-AKI development. Potential confounders (features that differed between the two groups) and variables with *P* < 0.1 in univariate analyses were included in the multivariate model. In multivariate analysis including HDL-C as quartiles rather than continuous variables, a significant linear trend was observed between the quartiles of HDL-C (quartile 1 vs. 2: odds ratio [OR], 0.716; 95% confidence interval [CI], 0.421–1.219; quartile 1 vs. 3: OR, 0.534; 95% CI, 0.301–0.947; quartile 1 vs. 4: OR, 0.173; 95% CI, 0.079–0.377; *P* for trend < 0.001) In addition, history of CVA (OR 2.023, 95% CI 1.142–3.587; *P* = 0.016), LVEF (OR 0.960, 95% CI 0.945–0.975; *P* < 0.001) and non-HDL-C (OR 1.006, 95% CI 1.001–1.010; *P* = 0.013) were independent variables for the development of CI-AKI (Table 2). HDL-C levels as quartiles were also negatively correlated with the incidence of CI-AKI whereas non-HDL-C levels as quartiles were not positively correlated with the incidence of CI-AKI (Fig. 1).

In multivariate Cox regression analysis of the long term mortality, the hazard ratio (HR) of HDL-C quartile 4, using the HDL-C quartile 1 as the reference category, was 0.516 (95% CI, 0.320–0.832, *P* = 0.007). In addition, age (HR 1.063, 95% CI 1.043–1.084; *P* < 0.001), LVEF (HR 0.963, 95% CI 0.952–0.975; *P* < 0.001), albumin (HR 0.516, 95% CI 0.320–0.832; *P* < 0.001) and CI-AKI (HR 1.063, 95% CI 1.043–1.084; *P* < 0.001) (Table 3).

**Figure 2:**
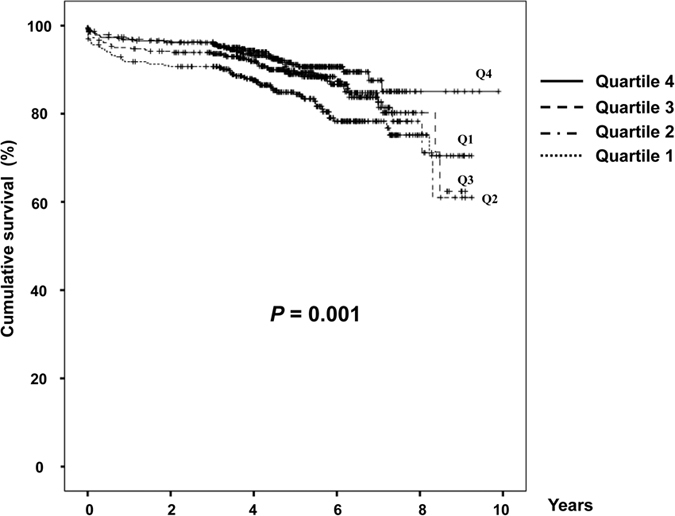
Kaplan-Meier plot of patient survival by HDL-C quartiles.

Figure 2 shows the Kaplan-Meier plot of patient survival by HDL-C quartiles. As shown, the survival was lower in the patients with the lowest HDL-C quartile compared with those with the highest HDL-C quartile (*P* = 0.001).

## Discussion

Our study showed a higher HDL-C level was associated with the decreased risk of CI-AKI and the better long term survival in CKD patients undergoing PCI.

The CKD population is known to be at higher risk of cardiovascular diseases, because their CKD state itself is associated with increased inflammation and oxidative stress, all of which are well known etiologies of atherosclerosis^12^,^13^. Percutaneous interventional procedures performed in this population are at higher risk of CI-AKI^4^. The contrast medium during the procedure is known to constrict renal arteries and decrease renal blood flow which contributes to ischemic insults in the kidneys. Concentrated stasis of iodinated contrast in renal tubules is also known to cause direct renal tubular toxicities and injuries. The resultant reactive oxygen species-mediated oxidative stress by these two mechanisms induces an inflammatory response in the kidneys, which may manifest as CI-AKI^14^,^15^,^16^. Hypotension or dehydration aggravates these ischemic renal insults, whereas N-acetylcysteine, which has been used for prevention of CI-AKI, is known to detoxify reactive oxygen species by acting as a free radical scavenger. CI-AKI may develop in patients with IHD undergoing coronary angiography and is known to be associated with poor clinical outcomes although the causes of CI-AKI can be multifactorial.

Dyslipidemia in CKD patients is characterized by dysregulation of the synthesis and activity of HDL-C and of the metabolism of triglyceride rich apolipoprotein B containing lipoproteins, which lead to the elevated plasma triglyceride and depressed HDL-C levels^17^. The combined oxidative stress, inflammation and dyslipidemia in CKD can accelerate atherosclerosis, the basic pathophysiology of all kinds of vascular disease. There is also a point of view that dyslipidemia makes significant contribution to CKD development suggesting that glomerulosclerosis and progressive glomerular and tubulointerstitial diseases are part of a spectrum of inter-related clinical disorders, including atherosclerosis, dyslipidemia, and oxidative and inflammatory stresses^18^,^19^. Several epidemiology studies have also documented an association between dyslipidemia and the CKD progression; the population with normal renal function who had low HDL-C and high non-HDL-C levels were at increased risk of CKD development^20^ whereas increased HDL-C levels were associated with a decreased risk of CKD progression^21^. Hypertriglyceridemia is also reported as an independent risk factor for developing CKD including proteinuria^22^,^23^.

It has not yet been established whether dyslipidemia per se is a risk factors for CI-AKI, despite the fact that several clinical studies have shown a benefit of pre-procedural statin in reducing CI-AKI^24^,^25^,^26^,^27^,^28^. The role of HDL-C in the development of CI-AKI is currently unknown. Only one study showed that a low HDL-C level was one of the significant risk factor of CI-AKI after PCI^29^. HDL-C, in addition to its well-known reverse cholesterol transport effect that lowers the total cholesterol level, has anti-oxidant, anti-inflammatory, and anti-apoptotic effects against the development and progression of atherosclerosis^30^,^31^. The functional integrity of HDL-C is as important as HDL-C level itself for its antiatherogenic effect. Some trials attempted to increase the HDL level by using torcetrapib, a cholesteryl ester transfer protein inhibitor, in which increased HDL levels were paradoxically linked to increased cardiovascular morbidities and mortalities^30^,^32^. Increasing these functional properties of HDL cannot be achieved by simply increasing its levels. Furthermore, recent studies suggest that systemic oxidative stress and inflammation in CKD substantially reduce the antioxidant and anti-inflammatory capabilities of HDL-C and even can convert HDL-C into a prooxidant and proinflammatory agent^17^,^33^.

Our study has limitations. First, the data were retrospectively analyzed, so unadjusted confounding factors may exist. Second, we showed that HDL cholesterol levels were inversely correlated with CI-AKI development without performing any functional assessment, despite the fact that recent studies have dealt with the quality of HDL cholesterol rather than its quantity.

In conclusion, low HDL-C level is associated with the increased risk of CI-AKI and long term mortality in the CKD patients undergoing PCI. Our study suggests that more intensive strategies including proper hydration, N-acetylcysteine use, exposure to less volume of contrast media, and careful monitoring of urine output and renal function, should be considered for preventing CI-AKI in CKD patients with low serum HDL-C level who is planned for PCI.

## Methods

### Study population

The study population was extracted from from the Catholic Medical Center percutaneous coronary intervention (COACT) registry, a prospective multicenter, all comer-based registry of patients undergoing PCI with drug eluting stents (DES) in 8 hospitals affiliated with the Catholic Medical Center in South Korea between January 1, 2004 to December 31, 2013. Among the 8334 patients with follow up laboratory data of more than 2 weeks duration after the index PCI, patients with end stage renal disease, those on hemodialysis or peritoneal dialysis (n = 426) and renal transplant recipients (n = 125) were excluded.

A total of 7783 patients were subsequently identified. In the patients with acute coronary syndrome (ACS), emergency coronary angiography(CAG) with PCI (within 2 hours) was performed in ST segment elevation MI (STEMI). In Non-STEMI (NSTEMI) and unstable angina, emergency CAG with PCI was also performed when refractory or recurrent angina, signs/symptoms of heart failure, new/worsening mitral regurgitation, hemodynamic instability, sustained ventricular tachycardia, or ventricular fibrillation was present. Otherwise, early (within 24 hours) or delayed invasive (within 25–72 hours) PCI was performed based on the discretion of the interventional cardiologist. Therefore the entire patient cohort can be divided into two groups consisting of emergency PCI (n = 2950) and non-emergency PCI (n = 4833). The non-emergency group was comprised of patients with ACS treated by early or delayed invasive PCI and patients diagnosed with stable angina who were electively treated by PCI. From this non-emergency PCI group, we extracted 1592 CKD patients who were diagnosed with stable angina and electively treated by elective PCI. CKD was defined as MDRD eGFR less than 60 mL/min/1.73 m^2^, the presence of albuminuria (urinary albumin-to-creatinine ratio greater or equal than 30 mg/g) or proteinuria (a spot urine protein-to-creatinine ratio [UPCR] greater or equal than 300 mg/g) for >3 months, which is the accepted cutoff value of CKD by the Kidney Disease Improving Global Outcomes (KDIGO) guidelines^34^. All patients received pre-procedure intravenous hydration with half normal saline at the rate of 1 mL/kg body weight per hour. After exposure to contrast medium, patients again received half normal saline intravenously at a rate of 1 mL/kg/h for 12 hours. This hydration rate was reduced in patients with decreased LVEF or overt heart failure. The contrast agent used for primary PCI was iodixanol, an isosmolar nonionic dimer (Visipaque; GE Healthcare, Princeton NJ, USA).The registry was approved by the Institutional Review Board of the Catholic Medical Center of Korea (IRB No.XC11RIMI0107K).

### Definition

CI-AKI was defined as an absolute increase in serum creatinine (SCr) by >0.3 mg/dL or a relative increase of SCr by >50%, in accordance with the Acute Kidney Injury Network criteria. CI-AKI was diagnosed based on pre- and post-PCI SCr measurements. The pre-PCI SCr level was defined as the level measured just before the procedure. The post-PCI SCr level was defined as the highest level measured within 7 days of PCI.

The MDRD eGFR (mL/min/1.73 m^2^) was calculated using the equation: eGFR = 175 × SCr (mg/dL)^−1.154^ × (age)^−0.203^ × (0.742 if female).

### Collection of clinical and laboratory data

Variables included in the analysis were: demographics (age, gender, height, and body weight [BW]), medical history (presence of DM, hypertension and CVA), smoking status, LVEF measured by echocardiography, laboratory findings; hemoglobin, albumin, phosphorus, uric acid, pre-procedural plasma glucose, UPCR, lipid profile including total cholesterol (TC), HDL-C, non-HDL-C and measured low density lipoprotein cholesterol (LDL-C) levels except triglyceride level because triglyceride level should be measured only in the fasting state, measured before PCI, and SCr levels before and after PCI measured during hospitalization, and contrast volume (CV) used during PCI. After hospital discharge, assessment of the survival status and validation of clinical outcomes were performed by collecting records from the outpatient clinic or by follow-up telephone interviews.

### Statistical analyses

Continuous variables are presented as mean ± SD and were compared using *t*-test for independent samples. Variables not normally distributed are presented as median and IQR and were compared with the Mann–Whitney *U* test. Categorical variables were compared using a chi-square test or Fisher’s exact test, as appropriate. Binary logistic regression for estimating the OR and 95% CI was used to identify the predictive factors for the development of CI-AKI. Survival curves were estimated using the Kaplan-Meier method and compared using the log-rank test. Cox proportional-hazard regression models were used to calculate hazard ratios (HRs) with 95% confidence intervals (CIs) for the long term mortality. Variables with a variance inflation factor greater than 10 were considered to have a multicollinearity problem. All p values were two-tailed, and *P* values < 0.05 were considered significant. Statistical analyses were performed using SPSS version 15.0 for Windows (SPSS, Chicago, IL, USA).

## Additional Information

**How to cite this article**: Park, H. S. *et al*. HDL Cholesterol Level Is Associated with Contrast Induced Acute Kidney Injury in Chronic Kidney Disease Patients Undergoing PCI. *Sci. Rep.* **6**, 35774; doi: 10.1038/srep35774 (2016).

## Figures and Tables

**Table 1:** Baseline characteristics of the groups with and without CI-AKI.

	Group with CI-AKI(N = 193)	Group without CI-AKI(N = 1399)	*P* value
Age (years)	72 (65–77)	69 (62–74)	<0.001
Female	86 (44.6%)	630 (45.0%)	0.9
BMI (kg/m^2^)	23.7 ± 2.9	24.8 ± 3.2	<0.001
DM	100 (51.8%)	584 (41.7%)	0.008
Hypertension	142 (73.6%)	957 (68.2%)	0.13
Smoking	54 (28.0%)	357 (25.5%)	0.464
History of CVA	29 (15.0%)	115 (8.2%)	0.002
LVEF (%)	58.9 (46.5–66.0)	61.0 (55.0–66.0)	<0.001
Hemoglobin (g/dL)	11.9 (10.2–13.6)	12.8 (11.5–14.0)	<0.001
Albumin (g/dL)	3.1 (2.6–3.6)	3.3 (3.0–3.8)	<0.001
Pre-procedural plasma glucose (mg/dL)	143.0 (112.5–214.0)	118.0 (100.0–157.0)	<0.001
TC (mg/dL)	172.0 (136.0–203.0)	171.5 (146.0–200.0)	0.586
HDL-C (mg/dL)	35.0 (29.0–41.0)	41.0 (34.0–49.0)	<0.001
Measured LDL C (mg/dL)	106.0 (78.3–135.5)	104.0 (81.0–130.0)	0.792
Non-HDL-C (mg/dL)	137.0 (103.0–164.3)	129.0 (105.0–157.0)	0.168
Uric acid (mg/dL)	6.5 (5.3–7.7)	5.9 (5.0–7.4)	0.013
Phosphorus (mg/dL)	5.0 ± 0.8	4.5 ± 0.8	<0.001
CV (mL)	253.0 (200.0–302.5)	256.0 (200.0–331.0)	0.234
Pre-procedural MDRD eGFR (mL/min/1.73 m^2^)	43.5 (34.0–55.1)	57.0 (47.1–63.6)	<0.001
UPCR (mg/g)	702.2 (355.2–1612.1)	734.9 (354.9–1457.5)	0.712

Data are mean ± standard deviation (SD), or numbers and percentages, or median (25th–75th percentile), as appropriate. CI-AKI, contrast-induced acute kidney injury; BMI, body mass index; DM, diabetes mellitus; CVA, cerebrovascular accident; LVEF, left ventricular ejection fraction; TC, total cholesterol; HDL-C, high density lipoprotein cholesterol; LDL-C, low density lipoprotein cholesterol; CV, contrast volume; MDRD eGFR, Modification of Diet in Renal Disease estimated glomerular filtration rate; UPCR, a spot urine protein-to-creatinine ratio.

**Table 2:** Multivariate logistic regression model using HDL-C quartile levels for predicting CI-AKI.

Variables	OR	95% CI	*P* value	*P* for trend
History of CVA	2.023	1.142–3.587	0.016	
LVEF	0.960	0.945–0.975	<0.001	
Non-HDL-C	1.006	1.001–1.010	0.013	
Pre-procedural HDL-C quartile 1			<0.001	<0.001
Pre-procedural HDL-C quartile 2	0.716	0.421–1.219	0.219	
Pre-procedural HDL-C quartile 3	0.534	0.301–0.947	0.032	
Pre-procedural HDL-C quartile 4	0.173	0.079–0.377	<0.001	
Pre-procedural MDRD eGFR	0.978	0.963–0.993	0.004	

*Adjusted for age, BMI, DM, hypertension, history of CVA, LVEF, hemoglobin, albumin, pre-procedural plasma glucose, HDL and non-HDL cholesterol, uric acid, phosphorus, pre-procedural MDRD eGFR and UPCR. *P* for trend refers to a linear trend across the lowest to highest quartile. Pre-procedural HDL quartile 1: <34 mg/dl, quartile 2: <40 mg/dl, quartile 3: <48 mg/dl, quartile 4: ≥48 mg/dl. HDL-C, high density lipoprotein cholesterol; CI-AKI, contrast-induced acute kidney injury; OR, odds ratio; CI, confidence interval; CVA, cerebrovascular accident; LVEF, left ventricular ejection fraction; MDRD eGFR, Modification of Diet in Renal Disease estimated glomerular filtration rate.

**Table 3:** Multivariate Cox regression analysis for long-term mortality.

Variables	OR	95% CI	*P* value
Age	1.063	1.043–1.084	<0.001
LVEF	0.963	0.952–0.975	<0.001
Albumin	0.516	0.320–0.832	<0.001
Pre-procedural HDL-C quartile 1			0.048
Pre-procedural HDL-C quartile 2	0.765	0.503–1.163	0.210
Pre-procedural HDL-C quartile 3	0.683	0.447–1.045	0.079
Pre-procedural HDL-C quartile 4	0.516	0.320–0.832	0.007
CI-AKI	1.063	1.043–1.084	<0.001

*Adjusted for age, DM, history of CVA, LVEF, hemoglobin, albumin, pre-procedural plasma glucose, HDL cholesterol, uric acid, phosphorus, UPCR and CI-AKI development. Pre-procedural HDL quartile 1: <34 mg/dl, quartile 2: <40 mg/dl, quartile 3: <48 mg/dl, quartile 4: ≥48 mg/dl. OR, odds ratio; CI, confidence interval; LVEF, left ventricular ejection fraction; HDL-C, high density lipoprotein cholesterol; CI-AKI, contrast-induced acute kidney injury.
